# Supplementary material for: Multivariate patterns linking brain microstructure to temperament and behavior in adolescent eating disorders
Source: Mol Psychiatry. 2025 Jul 16;30(11):5326–37. doi: 10.1038/s41380-025-03117-7 (PMC12282504; doi:10.1038/s41380-025-03117-7)
Supplement: Supplementary file 1 — Supplemental Material [file 41380_2025_3117_MOESM1_ESM.docx]

**SUPPLEMENTARY MATERIAL**

Multivariate patterns linking brain microstructure to temperament and behavior in adolescent eating disorders

Carolina Makowski^1^, Golia Shafiei^2^, Megan Martinho^1^, Donald J. Hagler Jr.^3^,

Diliana Pecheva^3^, Anders M. Dale^3,4,5^, Christine Fennema-Notestine^1,6^,

Amanda Bischoff-Grethe^1*^, Christina E. Wierenga^1*^

^1^Department of Psychiatry, University of California San Diego, La Jolla, California, USA

^2^Department of Psychiatry, Perelman School of Medicine, University of Pennsylvania, Philadelphia, Pennsylvania

^3^Center for Multimodal Imaging and Genetics, J Craig Venter Institute, La Jolla, California, USA

^4^University of California San Diego, La Jolla, California, USA

^5^Oslo University Hospital, Oslo, Norway

^6^Department of Radiology, University of California San Diego, La Jolla, California, USA

*Authors share senior authorship.

**Sample recruitment & inclusion criteria**

Female adolescents with an ED, meeting criteria for a DSM-5 restricting and binge-purge type eating disorder [1] [2], were recruited from the University of California, San Diego Eating Disorders Treatment and Research Program, as well as from the community. ED diagnosis was determined by meeting *DSM-5* criteria through the Kiddie Schedule for Affective Disorders and Schizophrenia (KSADS-5) [3, 4], a semi-structured interview performed by a trained research assistant, under the supervision of a doctoral level psychologist. HCs were recruited from the San Diego community. See Table 1 of the main manuscript for diagnostic breakdown of the ED group.

Exclusion criteria across individuals with an eating disorder and healthy controls (HC) included: history of alcohol or drug abuse or dependence 3 months prior to study, medical or neurological concerns including a history of head injury with loss of consciousness, and intellectual or developmental disability. Individuals with EDs were not excluded if they were taking psychoactive medications as long as the dose was stable (> 4 weeks). HCs were additionally excluded if they had any eating disorder symptoms, as determined by the Eating Disorders Examination (EDE) [5, 6], and/or a history of an eating disorder or any other Axis I disorder, as determined by the KSADS-5 [3, 4].

**MRI Acquisition.**

The imaging visit was scheduled during the early follicular phase (days 1 to 10) of the subject’s menstrual cycle, if she had begun (or resumed) menses. The imaging visit was conducted in the morning after an overnight fast, given the broader study protocol which included incentive processing of different taste stimuli. Self-report assessments relevant to this study (see Supplementary Table 1) were completed after the scan.

MRI Images were collected on a 3.0 T GE MR750 scanner equipped with quantum gradients providing echo planar capability, using a Nova Medical 32 channel head coil (maximum gradient strength: 50 mT/m, slew rate: 200 T/m/s). The following protocol was administered to acquire structural T1-weighted images and multi-shell diffusion: a three-plane localizer scan; a whole brain, sagittally acquired (0.8 mm slice thickness, FOV=256 mm) T1-weighted (MPRAGE PROMO, TE=3.656 ms, flip angle=8^o^, matrix=320, 2x in-plane acceleration) and separate T2-weighted (3D CUBE, 0.8 mm slice thickness, FOV= 256 mm, TE=60 ms, variable flip angle, matrix=320, 2x in-plane acceleration) sequence for alignment and morphometry; and two DTI scans (FOV=240 mm, slice thickness = 1.7 mm, matrix = 140 x 140, b=1500/3000 s/mm^2^; 102 diffusion directions) which were further used for restriction spectrum imaging (RSI) modeling, detailed in the section below.

*Image Quality Control*. During the scan, visual inspection of the images were combined with real time image reconstruction and online calculation of QC metrics. When compromised image quality was detected, the scan was stopped, and additional instructions were communicated with the participant to ensure compliance. After acquisition, images further underwent quality control procedures, adapted by the Human Connectome Project protocol. This included: 1) visual rating of the T1- and T2-weighted images; 2) visual assessment of DTI distortion; and 3) detection of motion artifacts. One hundred and five participants with an ED and 50 HC completed an MRI scan at baseline. Of this sample, 6 participants with an ED were not further processed due to failing the above quality control measures.

**MRI Processing Pipeline.**

Data were processed using the Adolescent Brain Cognitive Development^SM^ (ABCD^®^) Study pipeline for images acquired on a GE MR750 scanner, as described in [7]. All imaging measures relied on collection of T1-weighted (T1w) structural MRI images (1mm isotropic), which were acquired with a 3D T1w inversion prepared RF-spoiled gradient echo scan. Prospective Motion Correction with PROMO [8, 9] was applied in real-time to T1-weighted acquisitions. Although not applied to the diffusion-weighted images directly, this does enable more accuracy when registering our diffusion-weighted images to the T1-weighted scans. The PROMO approach uses a very brief “navigator” images embedded within the sMRI data acquisition, efficient image-based tracking of head position, and compensation for head motion [10]. The ABCD image processing pipeline [7] applied to the current dataset also incorporates various methods specific to diffusion MRI processing to help mitigate the impact of motion on processed images. Head motion is estimated by registering each frame to a corresponding image synthesized from a tensor fit, accounting for variation in image contrast across diffusion orientations [11]. Overall head motion is quantified as the average of estimated frame-to-frame head motion. Because abrupt head motion often results in severe signal loss in individual slices for a particular frame, a robust tensor fit is used to exclude these dark slices so that they do not influence the estimation of eddy current distortions. The dark slices are identified through standard linear estimation of tensor model parameters from log transformed images [12]. The root mean square (RMS) of the residual error for each frame of each slice is calculated across brain voxels and then normalized by the median RMS value across frames within a given slice. For a given slice, frames with normalized RMS greater than 3.2 are censored from subsequent tensor fits, resulting in a tighter fit for the non-censored frames. A total of three iterations are sufficient to settle upon a stable tensor fit excluding dark frames for a given slice (frame-slices). To prevent dark frame-slices from influencing the estimation of eddy current distortions, such frames are replaced (for a given slice) with the corresponding image synthesized from the censored tensor fit. Eddy Current Correction (ECC) is optimized using Newton’s method through minimization of RMS error between each eddy-current-corrected image and the corresponding image synthesized from the censored tensor fit, accounting for image contrast variation between frames. After applying corrections for the estimated distortions, we re-estimate the tensor, again excluding the dark frame-slices identified earlier, to produce a more accurate template for subsequent iterations of ECC, with five iterations in total. To correct images for head motion, we rigid-body-register each frame to the corresponding volume synthesized from the post-ECC censored tensor fit. We remove the influence of dark frame-slices from motion correction and future analysis by replacing those images with values interpolated from the tensor fit calculated without their contribution. The diffusion gradient matrix is adjusted for head rotation, which is important for accurate model fitting and tractography [11, 13]. After processing, an additional ED participant and 2 healthy controls failed the pipeline, leaving a final sample size of 98 participants with an ED and 48 healthy controls with high quality imaging data.

*Modeling of diffusion-weighted data with Restriction Spectrum Imaging (RSI).* We used RSI to model restricted directional diffusion (RND) across the entire brain. Details of this approach can be found in previous publications [10, 14, 15]. Briefly, RSI takes advantage of a multi-shell diffusion acquisition to estimate the contribution of diffusion signal from separable pools of water within a tissue, which includes free water (e.g., CSF), hindered diffusion (e.g., mostly extracellular space), and restricted diffusion (e.g., mostly intracellular space). Of interest to this study, restricted diffusion describes water within intracellular spaces confined by cell membranes with a non-Gaussian pattern of displacement. Spherical deconvolution (SD) is used to reconstruct the fiber orientation distribution (FOD) in each voxel from the restricted compartment, where the restricted tissue compartment is modeled as a fourth order spherical harmonic (SH) function. The restricted directional measure, RND, is the norm of the SH coefficients for the second and fourth order SH coefficients (divided by the norm of the coefficients across restricted, hindered, and free water compartments). In other words, RND models diffusion emanating from multiple directions within a voxel.

As a comparison to RND within superficial white matter, we also included fractional anisotropy (FA), derived from the diffusion tensor model [12, 16]. Diffusion tensor parameters were calculated using a standard, linear estimation approach with log-transformed diffusion-weighted (DW) signals [12]. Tensor matrices were diagonalized using singular value decomposition, obtaining three eigenvectors and three corresponding eigenvalues, from which FA could be calculated [16].

*Cortical thickness and volumes*. Cortical surfaces were constructed from T1-weighted structural images for each subject and segmented to calculate measures of cortical thickness along 5124 vertices using FreeSurfer v7.1.1 [17–21]. Cortical maps were smoothed using a Gaussian kernel of 20 mm full-width half maximum (FWHM) and mapped into standardized spherical atlas space. Processing with Freesurfer also yields automated tissue segmentation [22], which was used to determine volumes of subcortical structures (see section below).

**MRI-derived regions of interest.**

See Supplementary Tables 2-4 for a full list of ROIs used. For RND and FA analyses, 65 ROIs were used across 35 white matter tracts from the *AtlasTrack* probabilistic atlas [11] (Supplementary Table 2) and 30 subcortical structures from Freesurfer’s *aseg* atlas [22] (Supplementary Table 3). For morphometric analysis, cortical thickness across 68 regions from the *Desikan-Killiany* atlas [23] (Supplementary Table 4) and volumes for the above-mentioned 30 subcortical structures were included. Note, we retained ventricles and cerebrospinal fluid ROIs for RSI measures because it is possible to have restricted diffusion in ventricles if inflammatory markers are present. For instance, cerebral abscesses have been noted in patients with AN [24], which can cause diffuse patterns of abnormal restricted diffusion [25].

**Behavior**

Please see Supplementary Table 1 below for the behavioral instruments and variables included in analysis. Seven participants with an ED were dropped due to missing behavioral data in one or more of the instruments, resulting in our final sample reported in the main manuscript of 91 ED participants and 48 HC.

**Partial Least Squares model**

Partial Least Squares (PLS) is a form of reduced rank regression that identifies linear combinations of two sets of variables that maximally covary with each other [26, 27]. In the present study, one set represents neuroimaging-derived data for ED participants (denoted as​​ **X***_n_*_x_*_p_*), while the other set corresponds to the behavioral measures (denoted as **Y***_n_*_x_*_q_*). The *n* rows of both matrices **X** and **Y** represent the number of individuals with an ED (i.e., *n*=91). The *p* columns of matrix **X** correspond to the number of brain measures. For diffusion-based ROIs, we included 65 regions (35 white matter tracts, 30 subcortical structures); for the supplementary analyses with cortical thickness and volumes, 98 regions were included (68 cortical, 30 subcortical). The values in matrix **X** were then corrected for age using a linear regression model. The *q* columns of matrix **Y** correspond to the behavioral measures from the same ED participants, including 38 measures that capture clinical symptoms, cognition, temperament, and interoceptive awareness (See Supplementary Table 1 below). Finally, both **X** and **Y** matrices were standardized column-wise (i.e., z-scored) and a correlation matrix (**X’Y**) was computed from the standardized matrices. Singular value decomposition (SVD) was then applied to the correlation matrix **R=X’Y** as follows:

**R=X’Y = USV’**

The decomposition results in two orthonormal matrices of left and right singular vectors (**U** and **V**, respectively), and a diagonal matrix of singular values (**S**). The main results of PLS analysis are latent variables, which are weighted linear combinations of the original variables from the two initial variable sets (i.e., **X** and **Y**). PLS latent variables are mutually orthogonal and express the shared information between the two sets with maximum covariance. Latent variable *i* (LV*_i_*) is composed of the *i*th column vector of **U**, *i*th column vector of **V**, and the *i*th singular value from **S**. The elements of the column vectors of **U** and **V** are the weights of the original neuroimaging-derived values and behavioral measures, respectively, that contribute to the latent variable. The covariance between neuroimaging-derived and behavioral patterns is reflected in the corresponding singular values from the diagonal elements of matrix **S** and is estimated for the latent variable *i* as follows:

$$\eta_{i}=\frac{s_{i}^{2}}{\sum_{j=1}^{J} s_{j}^{2}}$$

where η*_i_* is the effect size for LV*_i_*, s*_i_* is the corresponding singular value from the diagonal matrix **S**, and *j* is the total number of singular values. Furthermore, the PLS-derived neuroimaging and behavioral patterns (i.e., left and right singular vectors, **U** and **V**) can be used to estimate individual-specific scores that reflect how much each individual expresses the PLS-derived patterns. The individual-specific brain and behavioral scores are calculated by projecting the neuroimaging and behavioral patterns (i.e., **U** and **V**) onto the original data:

Brain score = **XU**

Behavioral score = **YV**

We performed three additional analyses to assess the significance, reliability, and generalizability of the findings: (a) permutation testing to assess the statistical significance of the overall patterns; (b) bootstrap resampling to assess feature (imaging, behavioral) importance; (c) cross-validation analysis to assess the out-of-sample correlations between projected scores. Each step is discussed below.

*Permutation tests*. We assessed the statistical significance of each latent variable using permutation tests^63^ by randomizing the correspondence between brain and behavioral measures Specifically, PLS analysis was repeated after randomly reordering the rows of variable set **X** during each permutation. The procedure was repeated 10,000 times resulting in a null distribution of singular values. To test the null hypothesis that there is no relationship between imaging and behavioral measures, a *p*-value was estimated for each latent variable as the proportion of the times that the permuted singular values were greater than or equal to the original singular value.

*Bootstrap resampling*. The reliability of singular vector weights (i.e., weights of neuroimaging-derived values and behavioral variables) were assessed using bootstrap resampling (10,000 repetitions)^64^. The rows of the two variable sets (i.e., **X** and **Y**) were randomly resampled with replacement and PLS analysis was repeated with the new correlation matrix for each bootstrapped sample. This generated a sampling distribution for each neuroimaging and behavioral weight. To assess the reliability of each variable, bootstrap ratios were calculated as the ratio of each variable’s weight to its bootstrap-estimated standard error. Bootstrap ratios can be used to identify variables (brain regions of interest or behavioral measures) that make a large contribution to the overall pattern (i.e. have a large weight) and, at the same time, are stable across individuals (i.e. have a small standard error). If the bootstrap distribution is Gaussian, a bootstrap ratio can be interpreted as a z-score^64^, such that 95% and 99% confidence intervals correspond to bootstrap ratios of +1.96 and + 2.58, respectively.

*Cross-validation*. Cross-validation analysis was used to assess the out-of-sample correlation between brain and behavior scores^65,110^. We used 100 randomized train-test splits of the original data, where 75% of the data was treated as a training set and 25% of the data was treated as an out-of-sample test set. For each training set, PLS was used to estimate neuroimaging-derived and behavioral patterns (i.e., **U***_train_* and **V***_train_*). Importantly, the input neuroimaging data were not pre-residualized for age. Instead, we first regressed out age from brain and behavior scores in the train set using a linear regression model. To avoid any potential information leakage between train and test set due to covariates, we applied the regression coefficients from the train set to the unseen test set to account for age effects in the test data. The test data were then projected onto the neuroimaging-derived and behavioral patterns derived from the training set, to estimate individual-specific scores and their correlation for the test sample (i.e. *corr(***X***_test_***U***_train_*, **Y***_test_***V***_train_*)) . This procedure was repeated 100 times to generate a distribution of out-of-sample correlation coefficients. Finally, we used permutation tests (100 repetitions) to assess the significance of these out-of-sample correlation coefficients, where we randomly reordered rows of the original neuroimaging matrix and repeated the above procedure for each permutation. The procedure generated a null distribution of correlation coefficients between neuroimaging and behavioral scores in the test sample. A *p*-value was then calculated as the proportion of correlation coefficients that were greater than or equal to the mean original out-of-sample correlation coefficient.

**SUPPLEMENTARY TABLES**

| **Domain** | **Instrument** | **Variables** |
| --- | --- | --- |
| Cognition | Wechsler Abbreviated Scale of Intelligence (WASI) [28] | Similarities raw score  Matrix Reasoning raw score  Vocabulary (Vocab) raw score  Block Design (Block) raw score |
|  | Flanker task [29] | Flanker uncorrected score (Cognitive inhibition or “CogInhibition” in manuscript) |
|  | NIH Toolbox, Dimensional Change Card Sort [30] | Dimensional Change Card Sort, uncorrected score (Cognitive flexibility or “CogFlexibility” in manuscript) |
| Temperament | Temperament and Character Inventory (TCI) [31] | Self Transcendence  Cooperativeness  Self Directedness  Persistence  Reward Dependence  Harm Avoidance  Novelty Seeking |
|  | The Adult Temperament Questionnaire (ATQ) [32] | Inhibitory Control  Attentional Control  Activation Control  Effortful Control |
|  | Behavioral Inhibition and Behavioral Activation Scales (BISBAS)[33] | Behavioral Inhibition  Reward Responsiveness  Motivational Bias |
| Interoceptive awareness | Multidimensional Assessment of Interoceptive Awareness (MAIA) [34] | Trust  Body  Self Regulation (Self Reg)  Emotional Awareness (Emo Aware)  Attention Regulation  Not Worrying  Not Distracting  Noticing |
| ED symptoms | Eating Disorders Examination (EDE) [5, 6] | Weight Concern  Shape Concern  Eating Concern  Restraint |
|  | Eating Pathology Symptoms Inventory (EPSI) [35] | Excessive Exercise  Restricting  Purging  Binge Eating |
| Emotion | Difficulties in Emotion Regulation Scale (DERS) [36] | Difficulties in Emotion Regulation (Diff Emot Reg) Total Score |
|  | Toronto Alexithymia Scale (TAS) [37] | Alexithymia Total Score |

**Supplementary Table 1**. Behavioral and clinical measures. All variables reflect standard subscale scores obtained directly from the instrument, with the exception of Motivational Bias from the BISBAS. Motivational bias was calculated as the difference between reward responsiveness and inhibition, reflecting the dominance of reward vs punishment sensitivity [38]. Some subscales from the EPSI were omitted as they were already largely captured by the EDE (cognitive restraint, body dissatisfaction), and/or have not been shown to have a significant bearing on clinical course in adolescent girls (muscle building, obesity attitude).

| **Abbrev** | **Fiber Name** | **Connected Brain Regions** |
| --- | --- | --- |
| Fx | fornix | hippocampus & mammillary nuclei of hypothalamus |
| CgC | cingulate cingulum | cingulate gyrus & entorhinal cortex  (cingulate portion) |
| CgH | parahippocampal cingulum | cingulate gyrus & entorhinal cortex  (parahippocampal portion) |
| CST | corticospinal tract  (pyramidal tract) | motor cortex & spinal cord |
| ATR | anterior thalamic radiations | thalamus & frontal lobe |
| UNC | uncinate | inferior frontal lobe & anterior temporal lobe |
| ILF | inferior longitudinal fasciculus | occipital lobe & temporal lobe |
| IFO | inferior frontal occipital fasciculus | occipital lobe & frontal lobe |
| Fmaj | forceps major | left occipital cortex & right occipital cortex |
| Fmin | forceps minor | left prefrontal cortex & right prefrontal cortex |
| CC | corpus callosum | left cortex & right cortex |
| SLF | superior longitudinal fasciculus | temporal and parietal lobes & frontal lobe |
| tSLF | temporal superior longitudinal fasciculus (arcuate fasciculus) | temporal lobe & frontal lobe |
| pSLF | parietal superior longitudinal fasciculus | parietal lobe & frontal lobe |
| SCS | superior corticostriate | superior cortex & striatum |
| fSCS | frontal superior corticostriate | superior frontal cortex & striatum |
| pSCS | parietal superior corticostriate | superior parietal cortex & striatum |
| SIFC | striatal inferior frontal cortex tract | inferior frontal cortex & striatum |
| IFSFC | inferior frontal to superior frontal cortical tract | inferior frontal cortex & superior frontal cortex |

**Supplementary Table 2**. White matter tract regions of interest, available through the *Atlas Track* parcellation [7, 11], used for analyses with restricted normalized diffusion (RND) and fractional anisotropy (FA). All ROIs have separate labels for left and right hemispheres, except for corpus callosum, forceps major and forceps minor.

| **ROI name** | **Description** |
| --- | --- |
| Cerebral_WM | Cerebral white matter |
| Lateral-Ventricle | Lateral ventricle |
| Inf-Lat-Vent | Inferior lateral ventricle |
| Cerebellum-WM | Cerebellum white matter |
| Cerebellum-Cx | Cerebellum grey matter |
| Thalamus | Thalamus-proper |
| Caudate | Caudate |
| Putamen | Putamen |
| Pallidum | Pallidum |
| Hippocampus | Hippocampus |
| Amygdala | Amygdala |
| Accumbens | Accumbens Area |
| VentralDC | Ventral diencephalon |
| 3rd_Ventricle | Third ventricle |
| 4th_Ventricle | Fourth ventricle |
| Brain_Stem | Brain stem |
| CSF | Cerebrospinal fluid |

**Supplementary Table 3**. Subcortical regions of interest, available through the *aseg* parcellation in Freesurfer (Fischl et al., 2002), and used in analyses of RND, FA, and volumes. All structures are labeled on both hemispheres, except for the third and fourth ventricles, brainstem, and cerebrospinal fluid.

| **ROI name** | **Description** |
| --- | --- |
| bankssts | Banks superior temporal sulcus |
| caudalanteriorcingulate | Caudal anterior-cingulate cortex |
| caudalmiddlefrontal | Caudal middle frontal gyrus |
| cuneus | Cuneus cortex |
| entorhinal | Entorhinal cortex |
| fusiform | Fusiform gyrus |
| inferiorparietal | Inferior parietal cortex |
| inferiortemporal | Inferior temporal gyrus |
| isthmuscingulate | Isthmus-cingulate cortex |
| lateraloccipital | Lateral occipital cortex |
| lateralorbitofrontal | Lateral orbital frontal cortex |
| lingual | Lingual gyrus |
| medialorbitofrontal | Medial orbital frontal cortex |
| middletemporal | Middle temporal gyrus |
| parahippocampal | Parahippocampal gyrus |
| paracentral | Paracentral lobule |
| parsopercularis | Pars opercularis |
| parsorbitalis | Pars orbitalis |
| parstriangularis | Pars triangularis |
| pericalcarine | Pericalcarine cortex |
| postcentral | Postcentral gyrus |
| posteriorcingulate | Posterior-cingulate cortex |
| precentral | Precentral gyrus |
| precuneus | Precuneus cortex |
| rostralanteriorcingulate | Rostral anterior cingulate cortex |
| rostralmiddlefrontal | Rostral middle frontal gyrus |
| superiorfrontal | Superior frontal gyrus |
| superiorparietal | Superior parietal cortex |
| superiortemporal | Superior temporal gyrus |
| supramarginal | Supramarginal gyrus |
| frontalpole | Frontal pole |
| temporalpole | Temporal pole |
| transversetemporal | Transverse temporal cortex |
| insula | Insular cortex |

**Supplementary Table 4**. Cortical regions of interest, available through the *Desikan-Killiany* parcellation [23] and used in supplementary analyses with cortical thickness. All structures are labeled on both left and right hemispheres.

**SUPPLEMENTARY FIGURES**

**
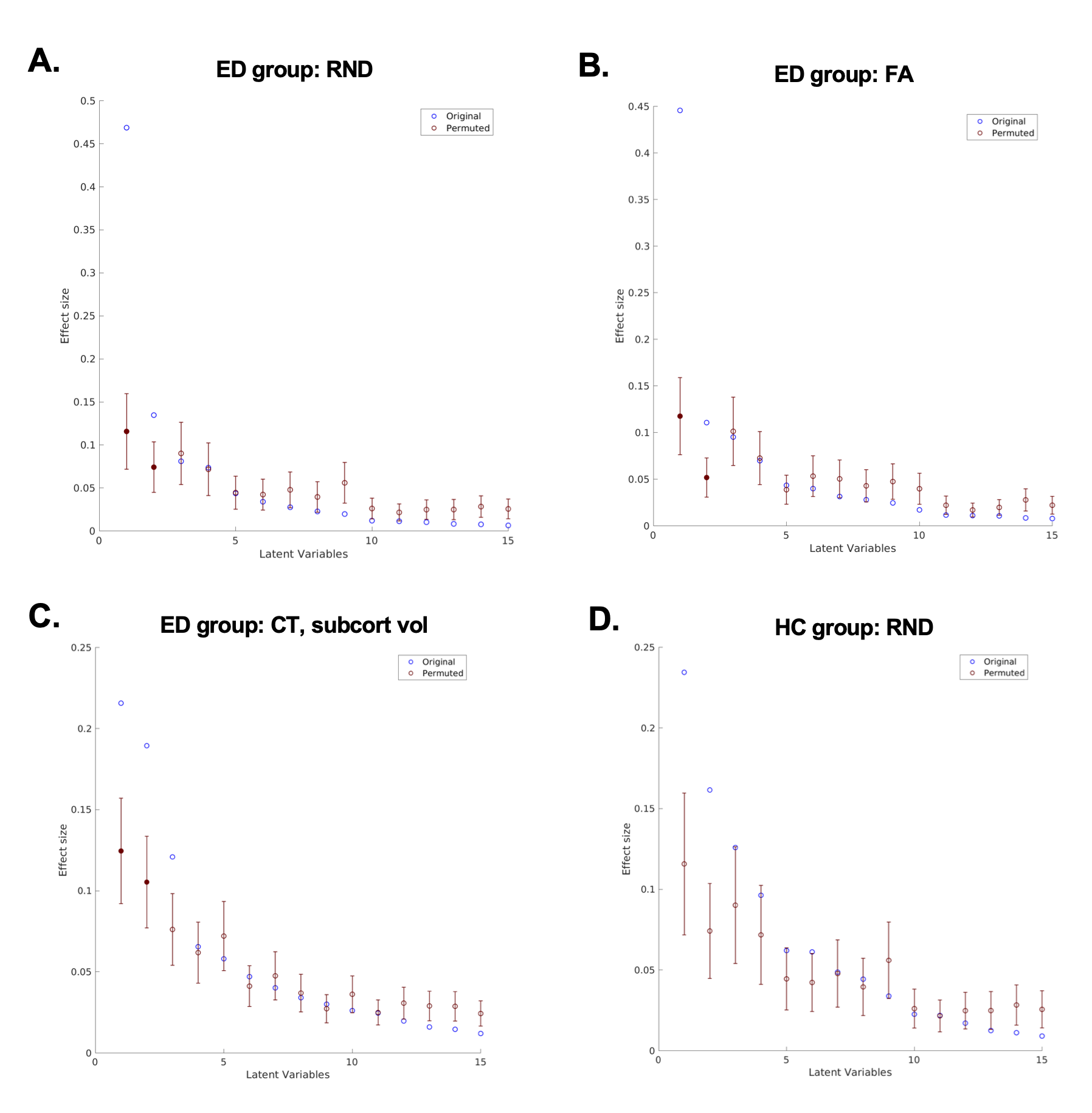
**

**Supplementary Figure 1**. Effect sizes (variance explained) across the top 15 LVs derived for each PLS analysis investigated in the current study. Filled red dots denote significant LVs, with a permuted *p*-value<0.05.

**
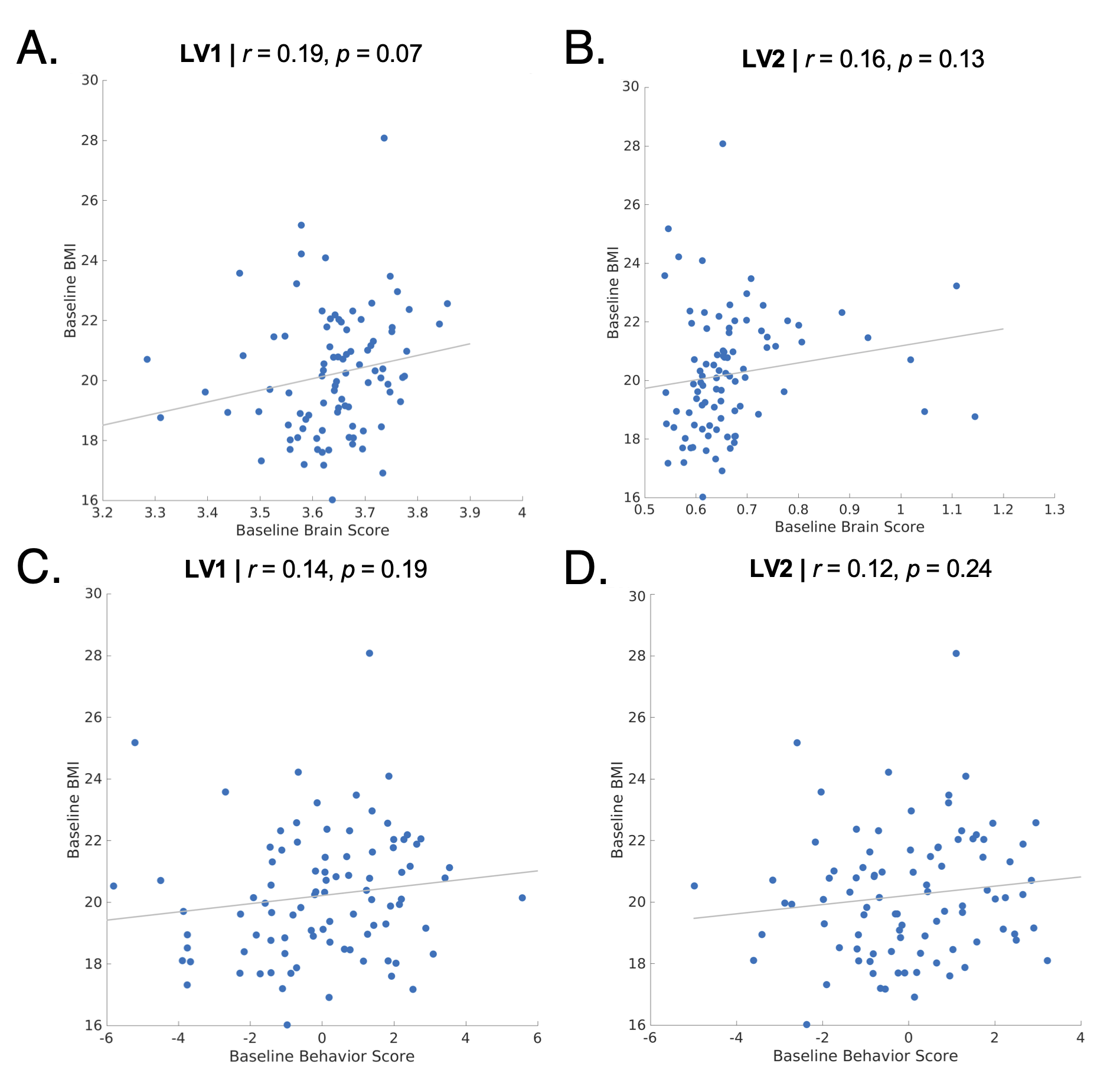
**

**Supplementary Figure 2**. No significant baseline BMI associations with Brain (Panel A: LV-1; Panel B: LV-2) or Behavior scores (Panel C: LV-1; Panel D: LV-2), derived from PLS analysis with RND brain measures.

**
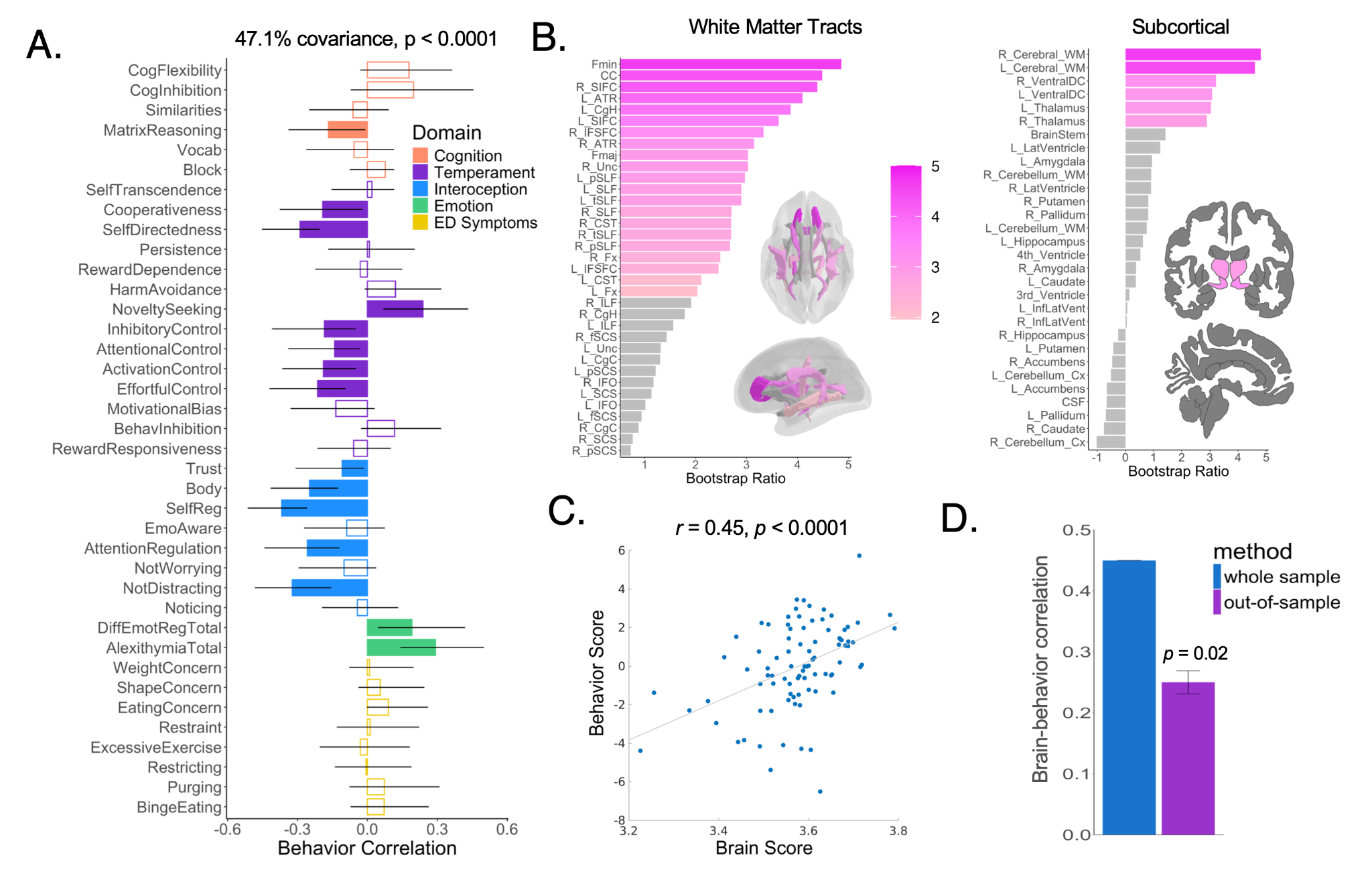
**

**Supplementary Figure 3. LV-1 results for brain measures derived from RND, dropping 3 ARFID participants.** Results are nearly identical to what is presented in the main manuscript in Figure 2. Panel A: Behavioral loadings, shown with correlation coefficients, of each included behavioral measure on LV-1. All loadings are color-coded by behavioral domain. Reliable loadings are portrayed with solid-filled bars, where error bars indicate bootstrap-estimated standard errors. Loadings with error bars crossing zero were interpreted as non-significant loadings and are portrayed by white-filled bars. Panel B: The contribution of RND within individual brain ROIs to LV-1, plotted as bootstrap ratios (ratios between ROI weights and bootstrap-estimated standard errors), which can be interpreted as z-scores. The gradient depicts bootstrap ratios for regions with a bootstrap ratio > |1.96| (corresponding to 95% confidence interval), whereas ROIs falling under this threshold are grey. See Supplementary Tables 2 and 3 for ROI abbreviations. Panel C: The projection of individual participant data onto each of the weighted patterns in Panels A and B shows that brain and behavior scores are positively correlated. This suggests that individuals who display the behavioral pattern in Panel A also tend to show increased FA in the significant brain regions in Panel B. Panel D: Correlations between brain-behavior scores in the full sample (same as Panel C) and in held-out data using the cross-validation scheme described in methods.

**
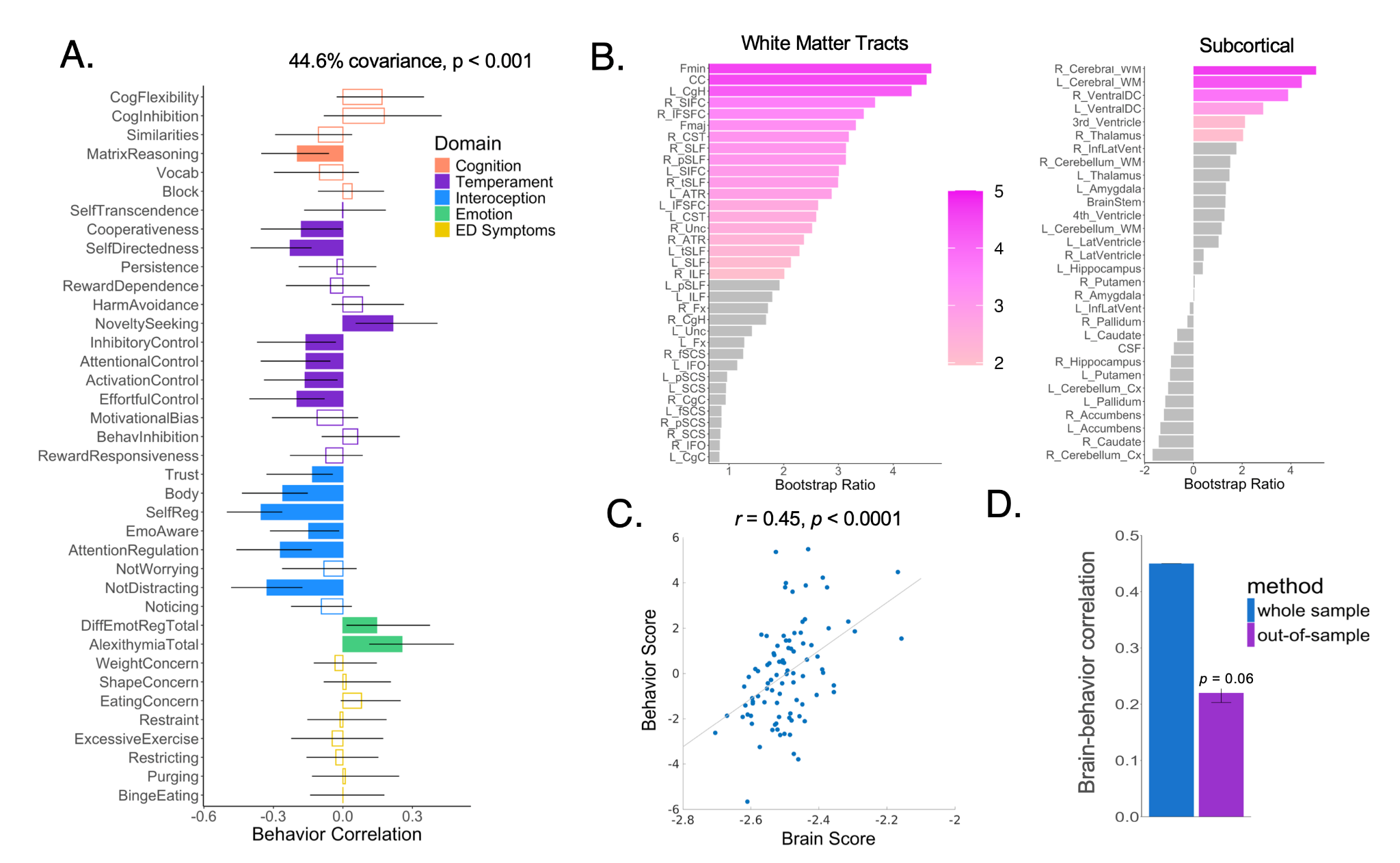
**

**Supplementary Figure 4.** LV-1 results for brain measures derived from FA. Panel A: Behavioral loadings, shown with correlation coefficients, of each included behavioral measure on LV-1. All loadings are color-coded by behavioral domain. Reliable loadings are portrayed with solid-filled bars, where error bars indicate bootstrap-estimated standard errors. Loadings with error bars crossing zero were interpreted as non-significant loadings and are portrayed by white-filled bars. Panel B: The contribution of FA within individual brain ROIs to LV-1, plotted as bootstrap ratios (ratios between ROI weights and bootstrap-estimated standard errors), which can be interpreted as z-scores. The gradient depicts bootstrap ratios for regions with a bootstrap ratio > |1.96| (corresponding to 95% confidence interval), whereas ROIs falling under this threshold are grey. See Supplementary Tables 2 and 3 for ROI abbreviations. Panel C: The projection of individual participant data onto each of the weighted patterns in Panels A and B shows that brain and behavior scores are positively correlated. This suggests that individuals who display the behavioral pattern in Panel A also tend to show increased FA in the significant brain regions in Panel B. Panel D: Correlations between brain-behavior scores in the full sample (same as Panel C) and in held-out data using the cross-validation scheme described in methods.


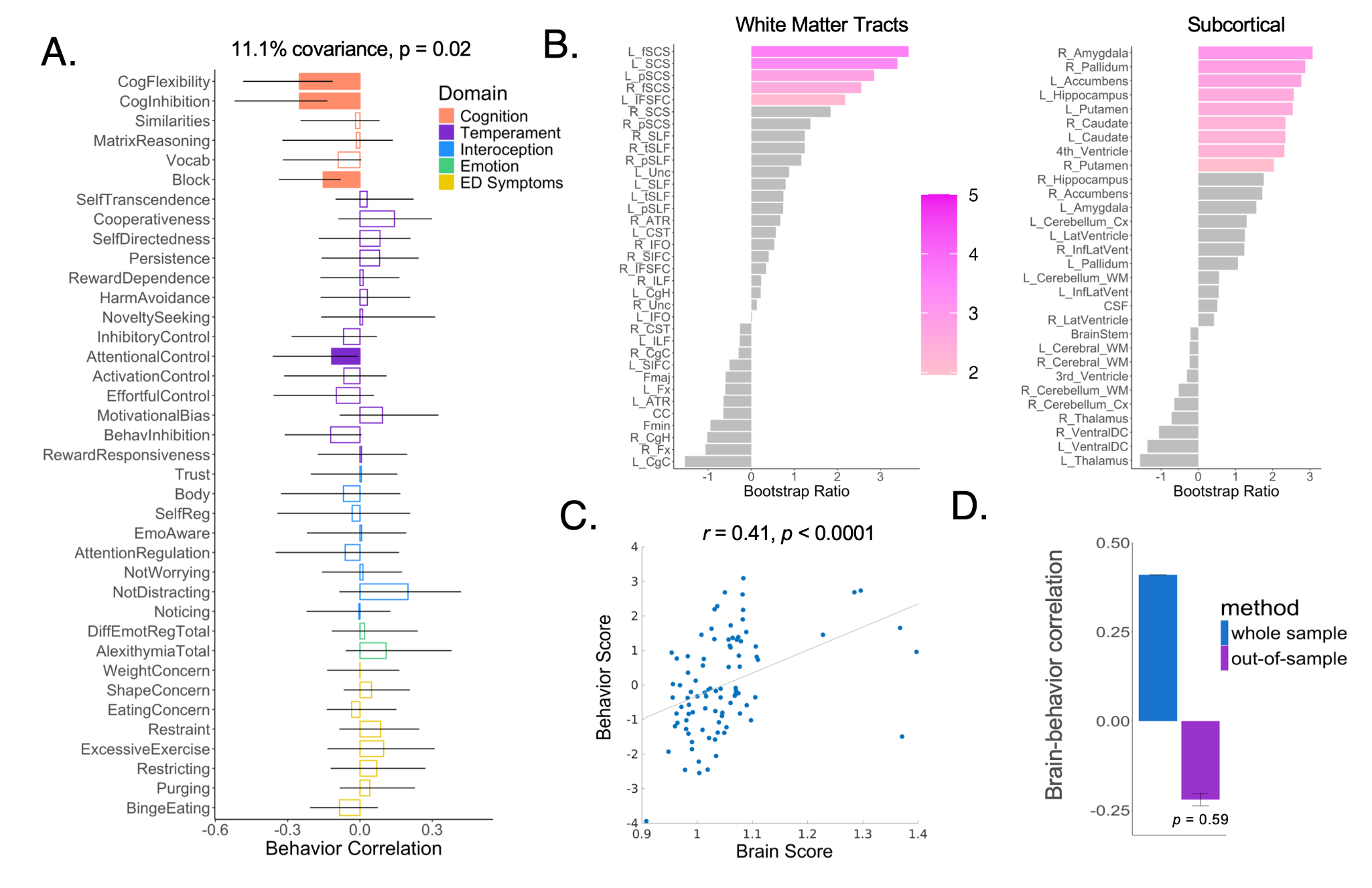


**Supplementary Figure 5.** LV-2 results for brain measures derived from FA. Panel A: Behavioral loadings, shown with correlation coefficients, of each included behavioral measure on LV-2. All loadings are color-coded by behavioral domain. Reliable loadings are portrayed with solid-filled bars, where error bars indicate bootstrap-estimated standard errors. Loadings with error bars crossing zero were interpreted as non-significant loadings and are portrayed by white-filled bars. Panel B: The contribution of FA within individual brain ROIs to LV-1, plotted as bootstrap ratios (ratios between ROI weights and bootstrap-estimated standard errors), which can be interpreted as z-scores. The gradient depicts bootstrap ratios for regions with a bootstrap ratio > |1.96| (corresponding to 95% confidence interval), whereas ROIs falling under this threshold are grey. See Supplementary Tables 2 and 3 for ROI abbreviations. Panel C: The projection of individual participant data onto each of the weighted patterns in Panels A and B shows that brain and behavior scores are positively correlated. This suggests that individuals who display the behavioral pattern in Panel A also tend to show increased FA in the significant brain regions in Panel B. Panel D: Correlations between brain-behavior scores in the full sample (same as Panel C) and in held-out data using the cross-validation scheme described in methods.

**
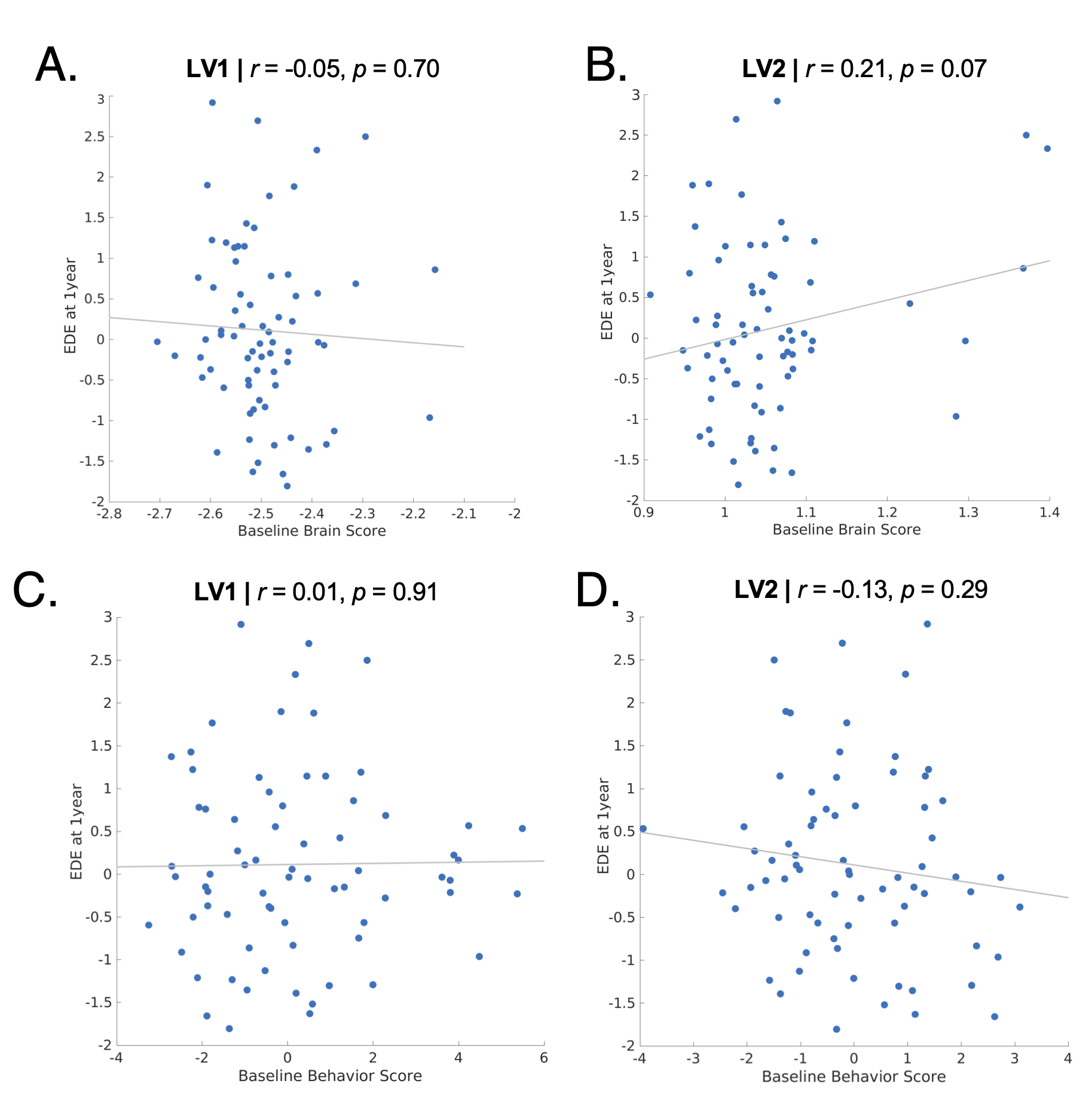
**

**Supplementary Figure 6.** Brain (Panel A: LV-1; Panel B: LV-2) and Behavior scores (Panel C: LV-1; Panel D: LV-2), derived from PLS analysis with FA brain measures, were not significantly associated with EDE scores one year later, after adjusting for baseline EDE scores.

**
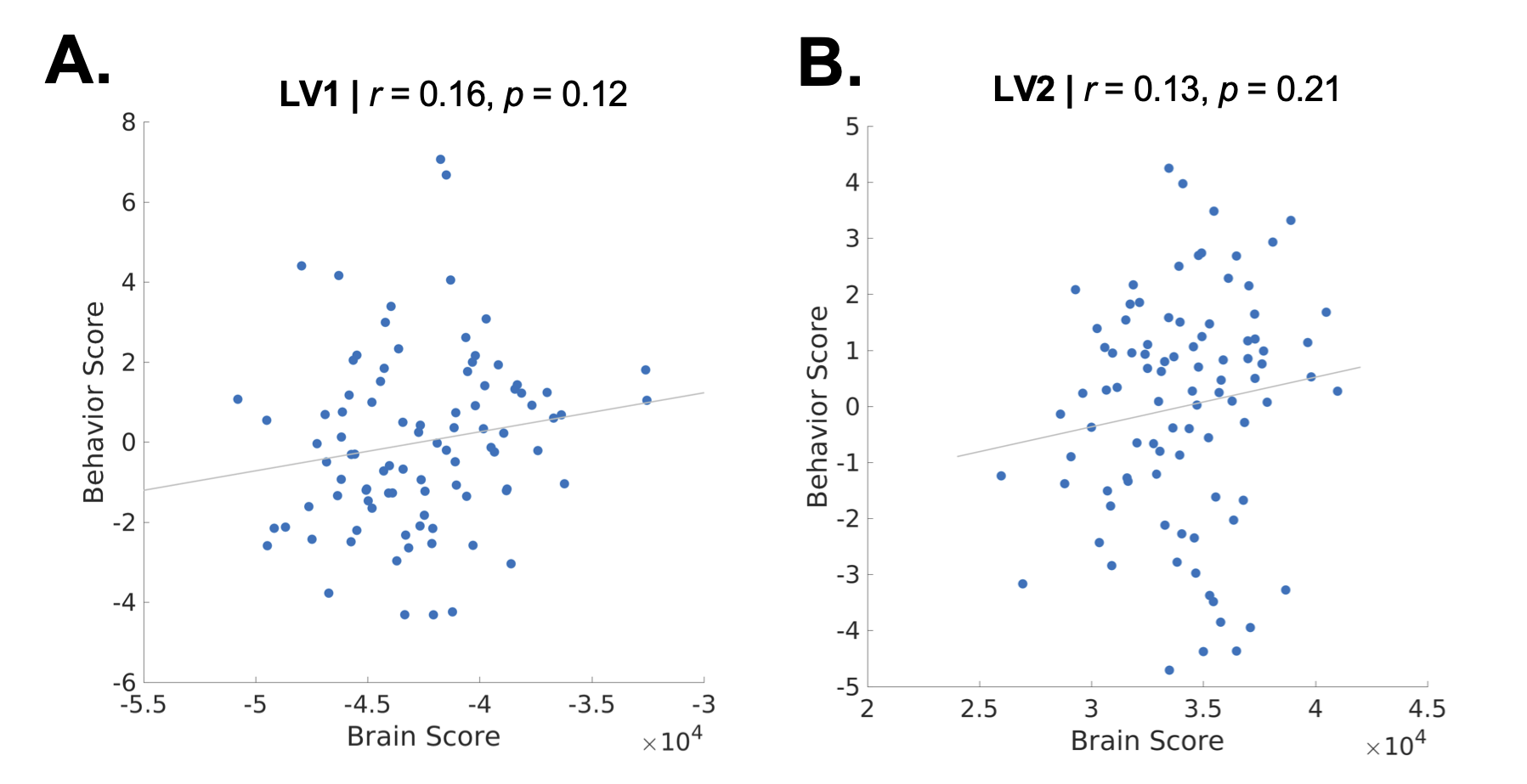
**

**Supplementary Figure 7.** Brain-behavior correlations for significant LVs (Panel A: LV-1; Panel B: LV-2) with cortical thickness/subcortical volume data used as input for the brain matrix for PLS. The top two LVs derived from these structural MRI features had significant permuted *p*-values, but did not show significant brain-behavior score associations. Further, these LVs did not survive permutation testing (LV1: out-of-sample *r*=-0.16, permuted *p*=0.07; LV2: out-of-sample *r*=-0.027, permuted *p*=0.61). Thus, these results were not further interpreted in the main manuscript.

**SUPPLEMENTARY REFERENCES**

1. Hebebrand J, Bulik CM. Critical appraisal of the provisional DSM-5 criteria for anorexia nervosa and an alternative proposal. Int J Eat Disord. 2011;44:665–678.

2. American Psychiatric Association. Diagnostic and Statistical Manual of Mental Disorders (DSM-5®). American Psychiatric Publishing; 2013.

3. Kobak KA, Kratochvil CJ, Stanger C, Kaufman J. Computerized screening of comorbidity in adolescents with substance or psychiatric disorders. Anxiety Disorders and Depression.

4. Kobak K, Kaufman J. Center for Telepsychology. 2015. 2015.

5. Fairburn CG, Beglin SJ. Assessment of eating disorders: interview or self-report questionnaire? Int J Eat Disord. 1994;16:363-370.

6. Mond JM, Hay PJ, Rodgers B, Owen C, Beumont PJV. Validity of the Eating Disorder Examination Questionnaire (EDE-Q) in screening for eating disorders in community samples. Behav Res Ther. 2004;42:551–567.

7. Hagler DJ Jr, Hatton S, Cornejo MD, Makowski C, Fair DA, Dick AS, et al. Image processing and analysis methods for the Adolescent Brain Cognitive Development Study. Neuroimage. 2019;202:116091.

8. White N, Roddey C, Shankaranarayanan A, Han E, Rettmann D, Santos J, et al. PROMO: Real-time prospective motion correction in MRI using image-based tracking. Magn Reson Med. 2010;63:91–105.

9. Brown TT, Kuperman JM, Erhart M, White NS, Roddey JC, Shankaranarayanan A, et al. Prospective motion correction of high-resolution magnetic resonance imaging data in children. Neuroimage. 2010;53:139–145.

10. White NS, Leergaard TB, D’Arceuil H, Bjaalie JG, Dale AM. Probing tissue microstructure with restriction spectrum imaging: Histological and theoretical validation. Hum Brain Mapp. 2013;34:327–346.

11. Hagler DJ Jr, Ahmadi ME, Kuperman J, Holland D, McDonald CR, Halgren E, et al. Automated white-matter tractography using a probabilistic diffusion tensor atlas: Application to temporal lobe epilepsy. Hum Brain Mapp. 2009;30:1535–1547.

12. Basser PJ, Mattiello J, LeBihan D. Estimation of the effective self-diffusion tensor from the NMR spin echo. J Magn Reson B. 1994;103:247–254.

13. Leemans A, Jones DK. Improved model fitting through improved eddy current distortion correction in DTI. Neuroimage. 2009;47:S51.

14. White NS, McDonald C, Farid N, Kuperman J, Karow D, Schenker-Ahmed NM, et al. Diffusion-weighted imaging in cancer: physical foundations and applications of restriction spectrum imaging. Cancer Res. 2014;74:4638–4652.

15. Palmer CE, Pecheva D, Iversen JR, Hagler DJ Jr, Sugrue L, Nedelec P, et al. Microstructural development from 9 to 14 years: Evidence from the ABCD Study. Dev Cogn Neurosci. 2022;53:101044.

16. Basser PJ, Pierpaoli C. Microstructural and physiological features of tissues elucidated by quantitative-diffusion-tensor MRI. J Magn Reson. 1996;213:560–570.

17. Dale AM, Fischl B, Sereno MI. Cortical surface-based analysis. I. Segmentation and surface reconstruction. Neuroimage. 1999;9:179–194.

18. Fischl B, Sereno MI, Dale AM. Cortical surface-based analysis. II: Inflation, flattening, and a surface-based coordinate system. Neuroimage. 1999;9:195–207.

19. Fischl B, van der Kouwe A, Destrieux C, Halgren E, Ségonne F, Salat DH, et al. Automatically parcellating the human cerebral cortex. Cereb Cortex. 2004;14:11–22.

20. Fischl B, Dale AM. Measuring the thickness of the human cerebral cortex from magnetic resonance images. Proc Natl Acad Sci U S A. 2000;97:11050–11055.

21. Jovicich J, Czanner S, Greve D, Haley E, van der Kouwe A, Gollub R, et al. Reliability in multi-site structural MRI studies: effects of gradient non-linearity correction on phantom and human data. Neuroimage. 2006;30:436–443.

22. Fischl B, Salat DH, Busa E, Albert M, Dieterich M, Haselgrove C, et al. Whole brain segmentation: automated labeling of neuroanatomical structures in the human brain. Neuron. 2002;33:341–355.

23. Desikan RS, Ségonne F, Fischl B, Quinn BT, Dickerson BC, Blacker D, et al. An automated labeling system for subdividing the human cerebral cortex on MRI scans into gyral based regions of interest. Neuroimage. 2006;31:968–980.

24. Yoshida K, Matsuda N, Sato T, Watanabe T, Nakamura K, Saito K, et al. Candida brain abscesses in a patient with anorexia nervosa receiving total parenteral nutrition. Clin Neurol Neurosurg. 2022;212:107058.

25. Benjamin P, Khan F, MacKinnon AD. The use of diffusion weighted imaging to evaluate pathology outside the brain parenchyma in neuroimaging studies. Br J Radiol. 2017;90:20160821.

26. McIntosh AR, Lobaugh NJ. Partial least squares analysis of neuroimaging data: applications and advances. Neuroimage. 2004;23 Suppl 1:S250–S263.

27. McIntosh AR, Mišić B. Multivariate statistical analyses for neuroimaging data. Annu Rev Psychol. 2013;64:499–525.

28. Wechsler D. Wechsler Abbreviated Scale of Intelligence: WASI. 1999.

29. Eriksen BA, Eriksen CW. Effects of noise letters upon the identification of a target letter in a nonsearch task. Percept Psychophys. 1974;16:143–149.

30. Zelazo PD. The Dimensional Change Card Sort (DCCS): a method of assessing executive function in children. Nat Protoc. 2006;1:297–301.

31. Cloninger CR, Svrakic DM, Przybeck TR. A psychobiological model of temperament and character. Arch Gen Psychiatry. 1993;50:975–990.

32. Chess S, Thomas A, Behavioral Developmental Initiatives. Adult Temperament Questionnaire. 1995.

33. Carver CS, White TL. Behavioral inhibition, behavioral activation, and affective responses to impending reward and punishment: The BIS/BAS Scales. J Pers Soc Psychol. 1994;67:319–333.

34. Mehling WE, Price C, Daubenmier JJ, Acree M, Bartmess E, Stewart A. The Multidimensional Assessment of Interoceptive Awareness (MAIA). PLoS ONE. 2012;7:e48230.

35. Forbush KT, Wildes JE, Pollack LO, Dunbar D, Luo J, Patterson K, et al. Development and validation of the Eating Pathology Symptoms Inventory (EPSI). Psychol Assess. 2013;25:859–878.

36. Gratz KL, Roemer L. Multidimensional assessment of emotion regulation and dysregulation: Development, factor structure, and initial validation of the difficulties in emotion regulation scale. J Psychopathol Behav Assess. 2004;26:41–54.

37. Bagby RM, Parker JD, Taylor GJ. The twenty-item Toronto Alexithymia Scale--I. Item selection and cross-validation of the factor structure. J Psychosom Res. 1994;38:23–32.

38. Abber SR, Murray SM, Brown CS, Wierenga CE. Change in motivational bias during treatment predicts outcome in anorexia nervosa. Int J Eat Disord. 2024;57:671–681.
